# Predicting pathological response of resectable esophageal squamous cell carcinoma to neoadjuvant anti-PD-1 with chemotherapy using serum inflammation indexes

**DOI:** 10.1038/s41598-025-11590-x

**Published:** 2025-07-31

**Authors:** Peng Song, Zhiyuan Yao, Shuai Song, Zengjin Wen, Xiao Sun, Changlei Li, Huansong Yang, Wenjie Jiao, Yong Cui, Dong Chang

**Affiliations:** 1 Department of Thoracic Surgery, Beijing Friendship Hospital, Capital Medical University, Beijing, China; 2 Department of Thoracic Surgery, The Affiliated Hospital of Qingdao University, Qingdao, China; 3 Key Laboratory of Carcinogenesis and Translational Research (Ministry of Education), Department of Thoracic Surgery II, Peking University Cancer Hospital & Institute, Beijing, China; 4 Department of Hepatobiliary and Pancreatic Surgery, The Affiliated Hospital of Qingdao University, Qingdao, China

**Keywords:** Esophageal squamous cell carcinoma, Neoadjuvant immunochemotherapy, Serum inflammation indexes, Prognosis, Pathological efficacy, Surgical oncology, Tumour biomarkers, Tumour immunology

## Abstract

**Background:**

Inflammatory indexes are increasingly being considered to predict treatment response in tumors. This study aimed to investigate the efficacy of serum inflammatory indexes in predicting pathological response in patients with esophageal squamous cell carcinoma (ESCC) receiving anti-PD-1 neoadjuvant immunochemotherapy (NICT).

**Methods:**

We retrospectively collected clinical and laboratory data from 116 ESCC patients who received NICT. We set three outcome variables: pathologic complete response (PCR), good response (GR), and response (R). We assessed between-group differences in inflammation indexes and their diagnostic efficacy. Independent diagnostic markers were filtered using least absolute shrinkage and selection operator (LASSO) logistic regression and multivariable analysis, and the corresponding nomograms for PCR and GR were constructed, respectively. Receiver operating characteristic curves (ROC) and calibration curves assessed the efficiency and accuracy of the models. Decision curve analysis (DCA) and clinical impact curves (CIC) evaluated the clinical value. Moreover, we internally validated the predictive model with a random sample of 30% of patients.

**Results:**

The prognostic nutritional index (PNI) predicted a cutoff value of 53.585 for PCR with an area under curve (AUC) value of 0.720, a cutoff value of 47.85 for GR with an AUC of 0.723, a cutoff value of 47.85 for R with an AUC of 0.629. Smoking and PNI were independent predictors of PCR, platelet-to-lymphocyte ratio (PLR) and PNI were independent predictors of GR, and PNI was an independent predictor of R. We built PNI-based nomograms to predict PCR and GR with AUC values of 0.795 and 0.763 for the training cohort and 0.907 and 0.757 for the validation cohort, respectively. The predicted and actual results of the calibration curves for both the training and validation groups showed good agreement, with Brier scores below 0.25.

**Conclusion:**

High PNI value is a shared independent predictor of achieving PCR, GR, and R in ESCC patients receiving anti-PD1 NICT. PNI-based diagnostic models can be used as a practical tool to identify ideal patients for personalized clinical decisions.

**Supplementary Information:**

The online version contains supplementary material available at 10.1038/s41598-025-11590-x.

## Introduction

Esophageal cancer is the sixth most common malignant tumor, with the seventh-highest mortality rate^1^. Esophageal cancer is often detected at a later stage due to the nonspecific nature of its early symptoms. To achieve tumor downgrading for surgery and optimal survival expectations, researchers have explored a variety of treatment modalities, including neoadjuvant chemotherapy, radiotherapy, immunotherapy, and combinations thereof^2^. The efficacy and safety of neoadjuvant immunotherapy have been demonstrated in clinical trials in multiple solid tumors, based on the principle that abundant tumor antigens exposure modulates the immune microenvironment, induces the clonal expansion of anti-tumor T-cells, and removes microscopic metastatic foci^3^. Besides, chemotherapeutic agents may enhance the effects of immunotherapy. For locally advanced esophageal squamous cell carcinoma (ESCC), neoadjuvant immunochemotherapy (NICT) demonstrated a superior pathological response to neoadjuvant chemoradiation therapy (NCRT)^4^ and neoadjuvant chemotherapy alone^5^. Mechanistically, single-cell sequencing revealed that chemotherapeutic agents activated cellular immunity^6^ and humoral immunity^7^ of ESCC patients, and the ensuing cytokine imbalance in the tumor microenvironment (TME) affected PD-1 expression. A clinical study found that appropriately increasing the dose of chemotherapeutic agents reduced PD-L1 resistance, increased the proportion of pathologic complete response (PCR), and improved disease-free survival (DFS) of locally advanced ESCC^8^.

In addition to PD-L1 and tumor mutation burden (TMB) as the most common potential predictors of ICI response, recent studies have found that M1 type tumor-associated macrophages, CD56dim NK cells^9^and macrophage migration inhibitory factor^10^ showed high predictive value for ESCC. However, technical limitations and prohibitive costs hindered their adoption.

Currently, inflammation indicators are widely used as immunotherapy efficacy biomarkers due to the routine availability of laboratory results to clinicians. Baseline inflammation scores before neoadjuvant therapy such as neutrophil-to-lymphocyte ratio (NLR), pan-immune-inflammation value (PIV), systemic immune-inflammatory index (SII), systemic inflammatory response index (SIRI), prognostic nutritional index (PNI) and platelet-to-lymphocyte ratio (PLR) predict pathological response of patients with multiple cancer types, such as breast cancer^11,12^gastric cancer^13^bladder cancer^14^and non-small cell lung cancer (NSCLC)^15^.

However, studies on the prediction of pathological response in ESCC patients treated with NICT remain limited. In this study, we retrospectively collected clinical data and peripheral blood indexes of ESCC patients receiving NICT in two hospitals from 2019 to 2023, intending to specifically explore the predictive value of inflammatory indexes on pathological responses of NICT for ESCC patients.

## Materials and methods

### Patients and treatment

We collected clinical and laboratory data of ESCC patients receiving anti-PD-1 NICT at Beijing Friendship Hospital and Affiliated Hospital of Qingdao University from June 2019 to December 2023. Patients were included in our study if meeting the following criteria: 1 Pathological diagnosis of ESCC; 2 Preoperative treatment with one to five cycles of anti-PD-1 inhibitors in combination with chemotherapeutic agents; 3 Undergoing radical esophagectomy. We excluded patients with the following characteristics: 1 Other induction therapy such as targeted therapy or radiotherapy, before or during the period of NICT. 2 Clinical signs of distant metastases. We obtained patients’ age, sex, history of smoking and alcohol, body mass index (BMI), PD-L1 expression status, types of anti-PD1 inhibitor and chemotherapeutic agents, total duration of NICT (NICT duration), interval from the last cycle of NICT to surgery (NICT-surgery interval), NICT cycles, and results of blood tests performed within the first one week before neoadjuvant therapy from electronic medical record system. Pathological findings were from postoperative resected tumor specimens. The Ethics Committee of The Beijing Friendship Hospital and The Affiliated Hospital of Qingdao University, approved this study protocol and all patients provided written informed consent prior to study commencement. The study was conducted in accordance to the Helsinki declaration.

The treatment regimen is intravenous anti-PD-1 inhibitors (200 mg of pembrolizumab, sintilimab, tirelizumab and camrelizumab, 240 mg of toripalimab, 300 mg of Nivolumab) combined with chemotherapy (Platinum + Paclitaxel/ Docetaxel/ Capecitabine/ Gemcitabine) every three weeks. Patients who have completed two to three cycles are reassessed to determine whether to continue induction or surgery. Patients who are suitable for surgery undergo thoracoscopic/robot-assisted or open radical esophagectomy within 4–6 weeks after completion of NICT.

### Pathological assessment after NICT

The HE-stained images of postoperative specimens were independently evaluated by two experienced pathologists at each institution using four categories of the American Joint Committee of Cancer and College of American Pathologists (AJCC/CAP) TRG system^16^. The grading is as follows: TRG0 (complete response), TRG1 (moderate response), TRG2 (minimal response), and TRG3 (poor response). TRG0 represents a pathological complete response (PCR). Due to the low percentage of patients achieving PCR (15/116), we developed two additional outcome indicators. We defined TRG0 and TRG1 jointly as Good-Response (GR) and TRG0, TRG1, and TRG2 as Response (R).

###  Calculation of serum inflammation indexes

Leukocytes, lymphocytes, platelets, monocytes, neutrophils, and albumin were recorded from the patient’s peripheral blood test results one week before receiving NICT, and the inflammatory indexes were calculated as follows: neutrophil -to-lymphocyte ratio (NLR), neutrophils (10^9^/L) / lymphocytes (10^9^/L)^17^; platelet-to-lymphocyte ratio (PLR), platelets (10^9^/L) / lymphocytes (10^9^/L)^18^; white-cell count-to-lymphocyte ratio (WLR), white-cell (10^9^/L) / lymphocytes (10^9^/L)^19^; pan-immune-inflammation value (PIV), neutrophils (10^9^/L) × platelets (10^9^/L) × monocytes (10^9^/L) / lymphocyte (10^9^/L)^20^; systemic immune-inflammatory index (SII), neutrophils (10^9^/L) × platelets (10^9^/L) / lymphocytes (10^9^/L)^21^; prognostic nutritional index (PNI), ALB (g/L) + 5 × lymphocytes (10^9^/L)^22^; systemic inflammatory response index (SIRI), neutrophils (10^9^/L) × monocytes (10^9^/L) / lymphocytes (10^9^/L)^23^.

### Statistical analysis

Statistical analyses were performed using R software (4.2.1) and SPSS (26.0). The least absolute shrinkage and selection operator (LASSO) model is highly effective in handling multivariable small sample data and solving the multicollinearity problem and is robust to outliers. Since multiple metrics are involved in lymphocytes, to address potential multicollinearity issues, we used the LASSO algorithm from the “glmnet” R package to narrow down the diagnostic candidates. LASSO regression models were cross-validated with the most predictive covariates selected for inclusion in multivariable analysis based on the lowest cross-validation error (λ min). According to multivariable analyses, we developed nomograms as diagnostic models to predict PCR and GR to determine the estimated probability of PCR and GR. Furthermore, we created ROC and calibration curves to assess the predictive accuracy of nomograms and produced decision curve analysis (DCA) and clinical impact curves (CIC) to confirm their clinical utility. The R packages used for multivariable analysis, building, and validating predictive models include “autoReg,” “AER,” “forestplot,” “rms,” “pROC,” and “rmda.” In addition, the “pROC” package is used to find the best cut-off value for each variable, with the “validation” package verifying its significance. Finally, we randomly selected 30% of all patients for internal validation of our models. Shapiro-Wilk and Levene’s Test were used to evaluate the normality and variance alignment of data, respectively. We use mean ± standard deviation or median (interquartile range) for continuous data and numbers (percentages) for categorical data. T-test was used for variables with a normal distribution and Mann-Whitney U-test was used for variables that did not conform to a normal distribution. The T-test/Mann-Whitney U-test was used for continuous data, while the Chi-square/Fisher’s exact test was applied for categorical data. Statistical significance was defined as a two-tailed * P*-value less than 0.05.

##  Results

### Clinical information

Patients’ basic clinical characteristics were presented in Table 1. A total of one hundred and sixteen patients were enrolled in this study, including one hundred and thirteen males and three females, with a mean age of 62.79 years. The majority of patients received two to four cycles of NICT, while three patients (2.6%) and one patient (0.9%) received one and five cycles, respectively. Nearly half of the patients (57, 49.1%) received the anti-PD1 inhibitor Sintilimab. More than half (61, 52.6%) were treated with the nedaplatin + paclitaxel regimen, followed by the carboplatin + paclitaxel regimen (43, 37.1%). Pathological TRG0, TRG1, TRG2, and TRG3 were recorded in 15 (12.9%), 25 (21.5%), 25 (21.5%) and 51 (44.0%) patients, respectively.

**Table 1:** Clinicopathological differences between efficacy groups.

Clinical characteristics	All patients (*n* = 116)	Patients with PCR (*n* = 15)	Patients with non-PCR (*n* = 101)	*P*	Patients with GR (*n* = 40)	Patients with non-GR (*n* = 76)	*P*	Patients with *R* (*n* = 65)	Patients with non-R (*n* = 51)	*P*
Age, X (SD)	62.79 (7.67)	64.13 (7.28)	62.59 (7.74)	0.47	64.47 (7.15)	61.91 (7.83)	0.087	63.94 (7.44)	61.33 (7.78)	0.069
BMI, X (SD)	22.66 (2.96)	22.93 (2.51)	22.62 (3.03)	0.705	22.69 (2.83)	22.65 (3.04)	0.95	22.69 (2.81)	22.63 (3.17)	0.919
Gender, n				0.343			0.273			1
Male	113	14	99		38	75		63	50	
Female	3	1	2		2	1		2	1	
Smoke, n				**0.011**			0.2			0.726
Yes	73	5	68		22	51		40	33	
No	43	10	33		18	25		25	18	
Wine, n				0.748			0.738			0.971
Yes	73	10	63		26	47		41	32	
No	43	5	38		14	29		24	19	
ypT, n				**< 0.001**			**< 0.001**			**< 0.001**
ypT0&T1	44	15	29		29	15		36	8	
ypT2&T3&T4	72	0	72		11	61		29	43	
ypN, n				0.46			0.069			**< 0.001**
ypN0&N1	97	14	83		37	60		61	36	
ypN2&N3	19	1	18		3	16		4	15	
PD-1 inhibitors, n				0.381			0.362			**0.022**
Pembrolizumab	13	1	12		5	8		9	4	
Tislelizumab	16	3	13		9	7		13	3	
Camrelizumab	21	5	16		6	15		7	14	
Sintilimab	57	5	52		18	39		33	24	
Others	9	1	8		2	7		3	6	
Chemotherapeutic agent, n				0.405			0.883			0.986
Carboplatin + Paclitaxel	43	5	38		14	29		24	19	
Nedaplatin + Paclitaxel	61	7	54		21	40		34	27	
Others	12	3	9		5	7		7	5	
Surgical procedures, n				0.191			0.19			0.101
Open	24	1	23		11	13		17	7	
Endoscope	92	14	78		29	63		48	44	
Cycles, M(QR)	2.00 (1)	2.00 (1)	2.00 (1)	0.426	2.00 (1)	2.00 (1)	0.409	2.00 (1)	2.00 (1)	0.638
Time1, M(QR)	29.00 (24)	27.00 (29)	29.00 (21)	0.905	29.00 (26)	28.50 (21)	0.238	29.00 (25)	28.00 (22)	0.393
Time2, M(QR)	34.00 (14)	39.00 (11)	34.00 (13)	0.11	37.00 (14)	33.50 (13)	0.113	34.00 (15)	34.00 (10)	0.670
PD-L1				1			0.533			0.239
< 10%	25	1	24		7	18		10	15	
≥ 10%	19	1	18		7	12		11	8	

The bold values denote statistically significant two-sides* P*-value < 0.05.

PD-L1 expression data of 44 ESCC patients who had performed PD-L1 immunohistochemistry was included in our study. Among the 44 patients, 19 (43.2%) had PD-L1 TPS ≥ 10% and 25 (56.8%) had PD-L1 TPS <10%. However, no significant association with treatment response was observed (PCR: *p* = 1; GR: *p* = 0.533; R: *p* = 0.239). Correlation analysis showed that PD-L1 expression was significantly correlated with PIV, PLR, and SII. However, PD-L1 expression had no correlation with PNI, NLR, SIRI and WLR (Supplementary Fig. 1). It is worth noting that: (1) Of all fifteen patients who achieved PCR, only five (33.3%) had smoking history; (2) Anti-PD-1 inhibitor Tislelizumab has significantly higher immunotherapy response rates than other drugs (81.3% vs. 52%, *p* = 0.028). (3) Gender, BMI, age, alcohol consumption history, chemotherapy regimen, induction therapy cycles, NICT duration and NICT-surgery interval were similar in both efficacy groups.

### The landscape of inflammation indexes between distinct groups based on PCR, GR, and R

**Fig. 1:**
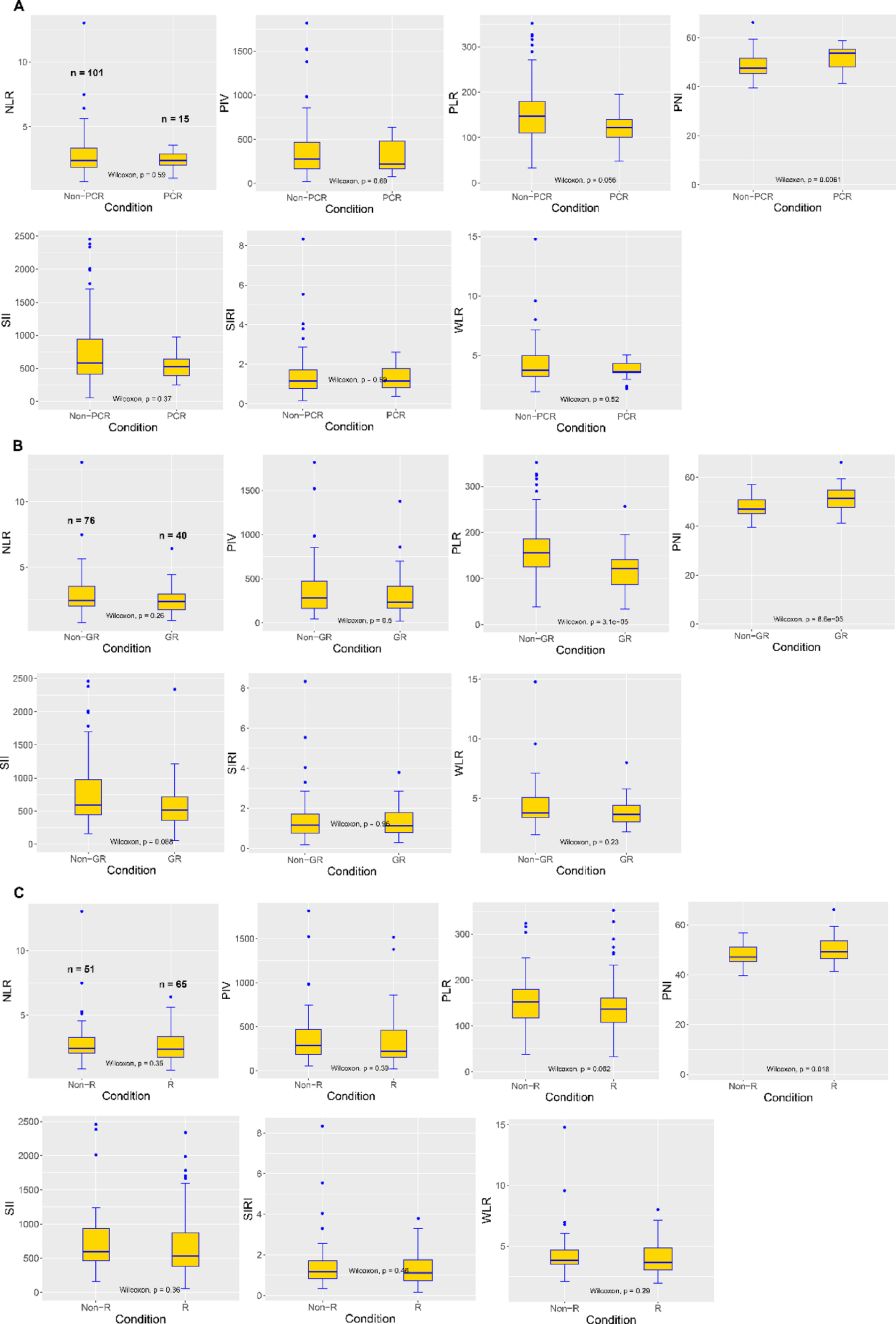
Differences in the distribution of each inflammatory indicator in the distinct response groups of PCR (**A**), GR (**B**), and R (**C**). PCR, pathologic complete response; GR, good response; R, response.

The distribution of NLR, PIV, SII, SIRI, and WLR was similar in patients who achieved PCR, GR, or R versus those who did not (Fig. 1). PLR in the PCR/GR group was significantly lower than that in the non-PCR/GR group (PCR: average value 122.61 vs. 153.81, *P* = 0.056; GR: average value 119.35 vs. 165.79, *P* < 0.001, respectively), whereas the PNI was significantly higher than that in the non-PCR/GR group (PCR: average value 51.92 vs. 48.46, *P* = 0.0061; GR: average value 51.49 vs. 47.55, *P* < 0.001, respectively). Only PNI was significantly higher in the Response group than in the non-response group (average value 49.98 vs. 47.54, *p* = 0.018).

### 3.3 Diagnostic efficacy of inflammatory indexes for PCR/GR/R

**Fig. 2:**
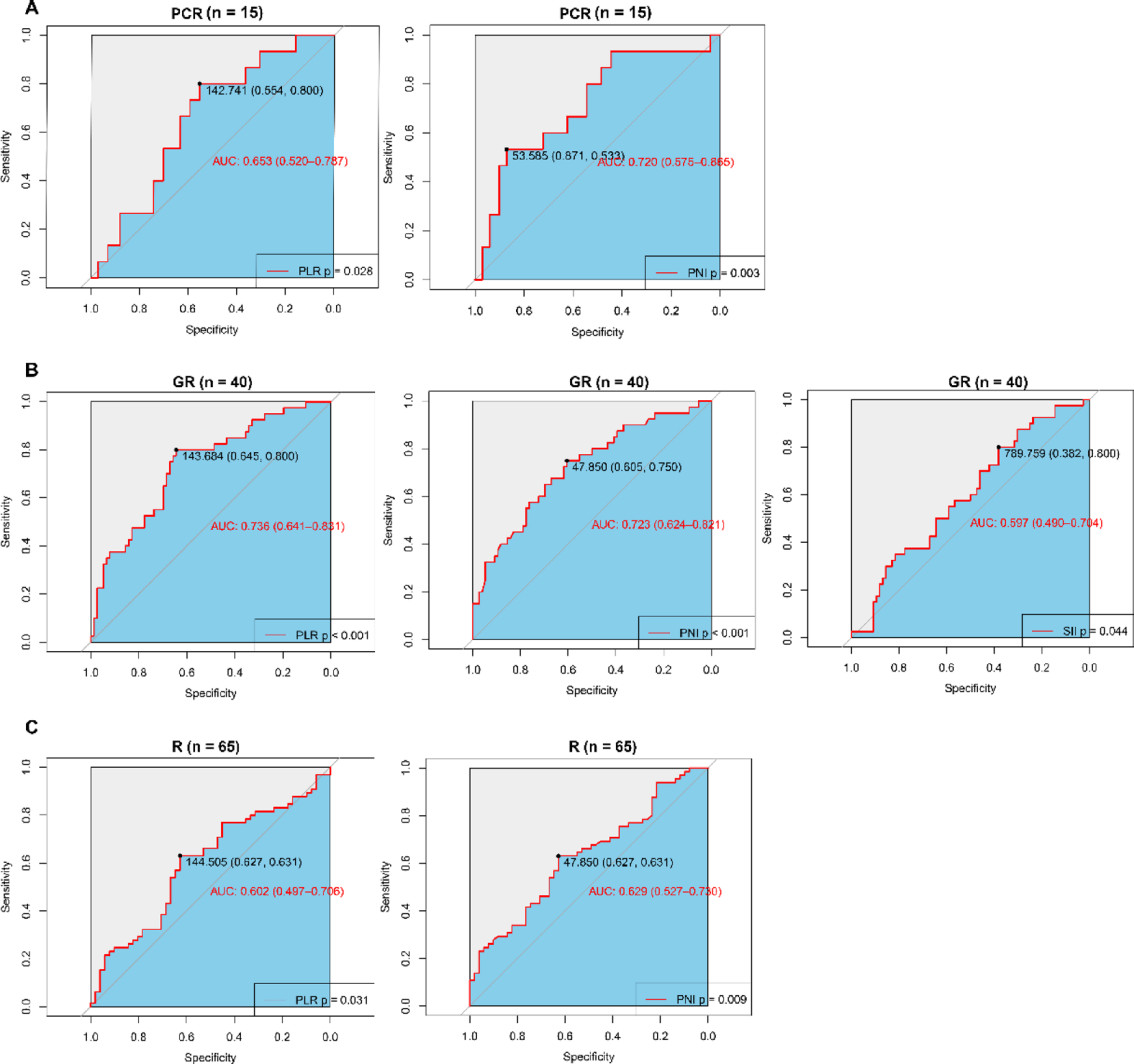
The prediction ability of statistically significant inflammatory indicators for PCR (**A**), GR (**B**), and R (**C**). PCR, pathologic complete response; GR, good response; R, response.

Figure 2 and Supplementary Fig. 2 showed the diagnostic efficacy of each inflammatory indicator for PCR, GR, and R. Interestingly, both PLR (PCR: cutoff = 142.741, AUC = 0.653, 95% CI 0.520–0.787, *P* = 0.028, sensitivity = 0.554, specificity = 0.800; GR: cutoff = 143.684, AUC = 0.736, 95% CI 0.641–0.831, *P* < 0.001, sensitivity = 0.645, specificity = 0.800; R: cutoff = 144.505, AUC = 0.602, 95% CI 0.497–0.706, *P* = 0.031, sensitivity = 0.627, specificity = 0.631) and PNI (PCR: cutoff = 53.585, AUC = 0.720, 95% CI 0.575–0.865, *P* = 0.003, sensitivity = 0.871, specificity = 0.533; GR: cutoff = 47.85, AUC = 0.723, 95% CI 0.624–0.821, *P* < 0.001, sensitivity = 0.605, specificity = 0.750; R: cutoff = 47.85, AUC = 0.629, 95% CI 0.527–0.730, *P* = 0.009, sensitivity = 0.627, specificity = 0.631) have good predictability for that three outcomes. In addition, SII has a significant predictive value for GR in ESCC patients (cutoff = 789.759, AUC = 0.597, 95% CI 0.490–0.704, *P* = 0.044, sensitivity = 0.382, specificity = 0.800). None of the remaining indexes were meaningful in predicting pathological reactions in our cohorts (Supplementary Fig. 2).

### Screening of diagnostic markers and creation and validation of nomograms

**Fig. 3:**
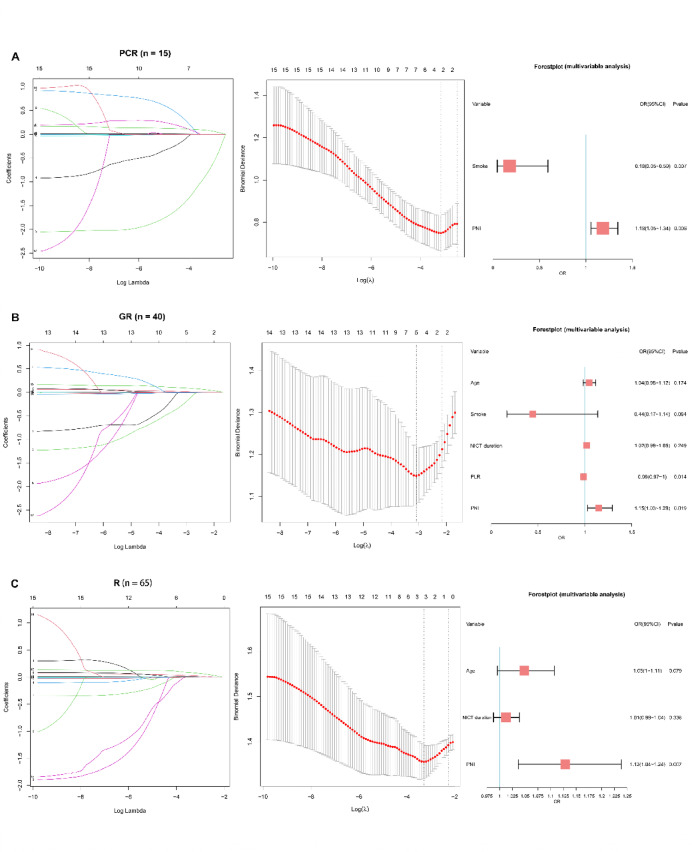
LASSO regression analysis and multivariable analysis of selected variables to screen independent diagnostic biomarkers to predict PCR (**A**), GR (**B**), and R (**C**) with cross-validation. LASSO, least absolute shrinkage and selection operator; PCR, pathologic complete response; GR, good response; R, response.

In light of the potential multicollinearity among inflammation indicators, we used LASSO regression analysis and cross-validation to screen the independent variables (Fig. 3). LASSO analyses included patients’ gender, age, history of smoking and alcohol, BMI, neoadjuvant cycles, NICT duration, NICT-surgery interval, NLR, PLR, SII, SIRI, PIV, WLR, and PNI. For PCR, λ min was 0.042, and the regression model included predictors smoke and PNI; For GR, λ min was 0.046, and the regression model included predictors age, smoke, NICT duration, PLR, and PNI; For R, λ min was 0.037, and the regression model included predictors age, NICT duration, and PNI. After multivariable analysis of the screened variables, we found that smoke and PNI were independent predictors of PCR; PLR and PNI were independent predictors of GR; PNI was the only independent predictor of R (Fig. 3).

**Fig. 4:**
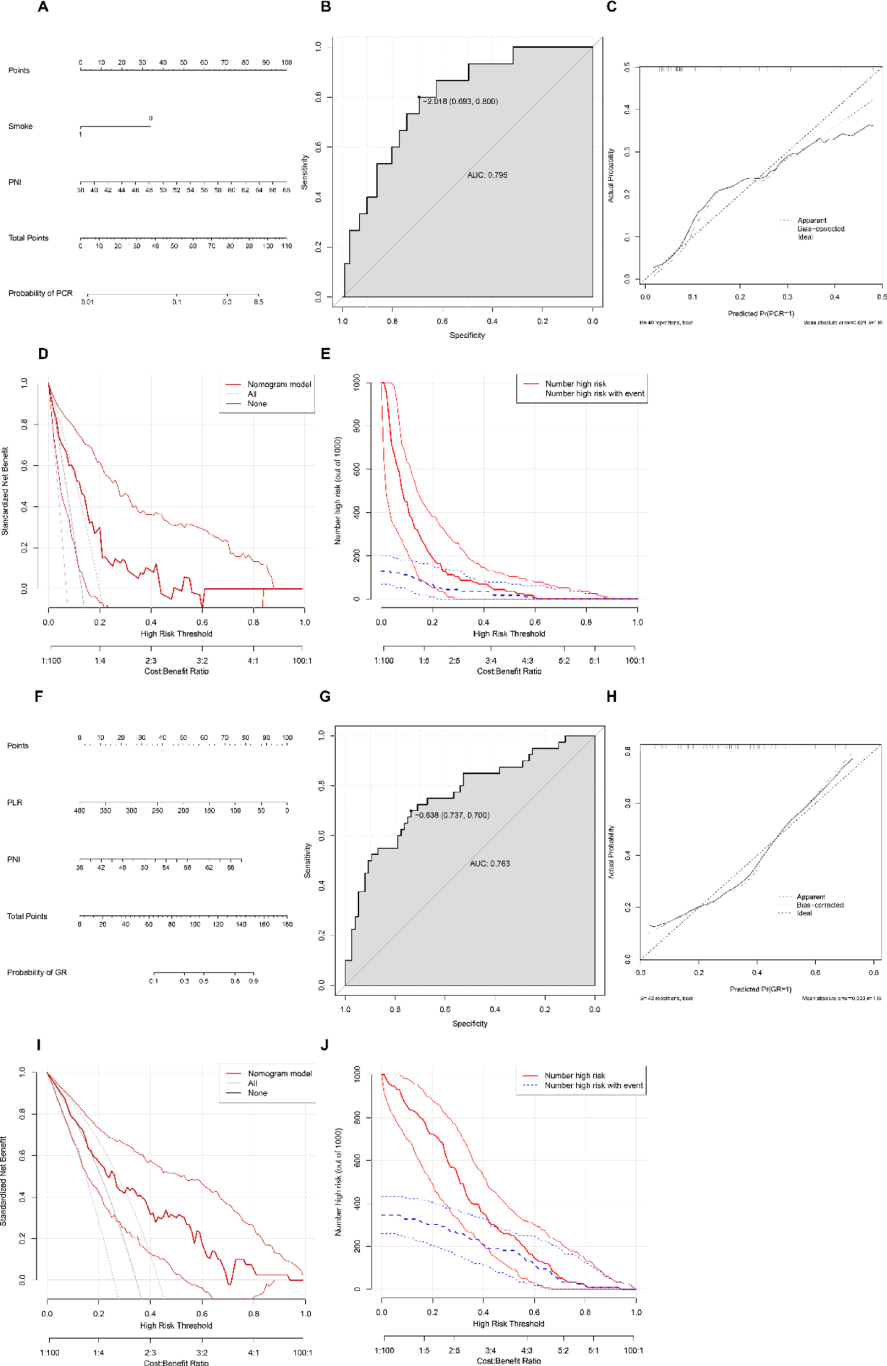
Development of the nomograms for PCR (**A**–**E**) and GR (**F**–**J**). (**A**,** F**) The nomograms for predicting PCR and GR for ESCC patients receiving NICT. The scores for each variable are summed to give a total score. Draw a perpendicular line on the point corresponding to the total score, which corresponds to the estimated probability. (**B**,** G**) ROC for predicting PCR and GR. (**C**,** H**) Calibration plots for the nomogram. The X and Y axes correspond to the predicted and actual probabilities of the model, respectively. The slope of the solid line is close to 45 degrees, indicating the accuracy of the model. The decision curve analysis (**D**,** I**) and clinical impact curves (**E**,** J**) for the nomograms indicate perfect clinical benefits for patients within a wide range of risk (probability) thresholds for predicting PCR and GR. PCR, pathologic complete response; GR, good response; R, response. ESCC, esophageal squamous cell carcinoma. NICT, neoadjuvant immunochemotherapy. ROC, receiver operating characteristic curves.

**Fig. 5:**
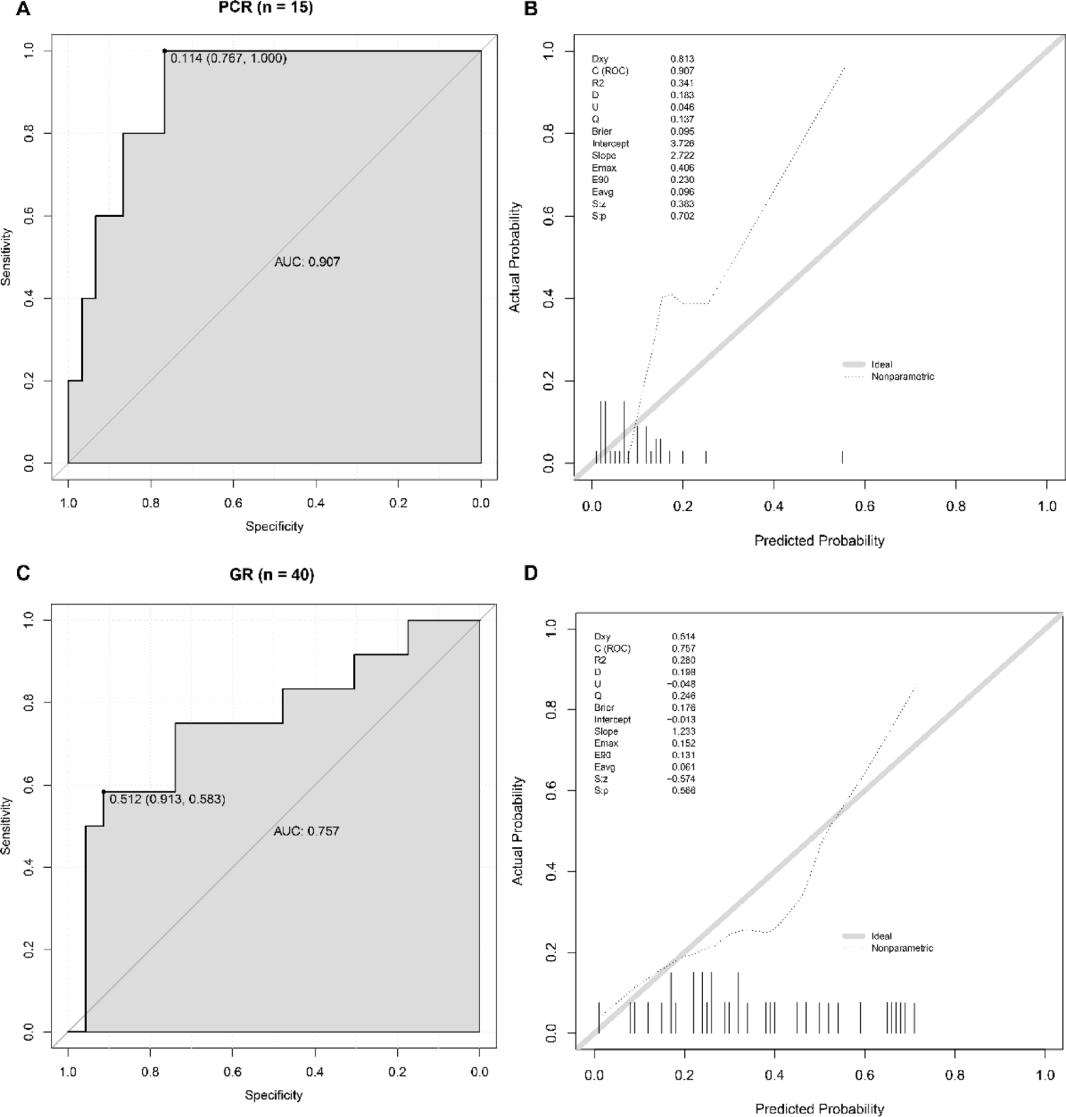
Validation of the nomograms for PCR (**A**,** B**) and GR (**C**,** D**). 30% of patients were randomly selected. (**A**,** C**) ROC for predicting PCR and GR in validation cohorts showed an excellent reliability of models. (**B**,** D**) Calibration plots showed good agreement of predicted and actual results, with Brier scores below 0.25. PCR, pathologic complete response; GR, good response; R, response.

Combining the above predictors, we produced diagnostic nomograms to predict PCR and GR of ESCC patients, respectively (Fig. 4). Surprisingly, it demonstrated that the nomogram combining smoke and PNI is an excellent predictive model for PCR (AUC = 0.795,95% CI 0.687–0.903, *P* < 0.001, sensitivity = 0.693, specificity = 0.800, Fig. 4A-B) and that combining PLR and PNI is an excellent predictive model for GR (AUC = 0. 763, 95% CI 0.669–0.857, *P* < 0.001, sensitivity = 0.737, specificity = 0.700, Fig. 4F-G). The calibrations demonstrated that the expected PCR and GR probabilities coincide excellently with the observed values, which indicates that the model has adequate accuracy (Fig. 4C and H). Moreover, DCA and CIC proved that ESCC patients receiving NICT can derive a net benefit from our diagnostic models (Fig. 4D-E and I-J). Finally, we randomly selected 30% of the patients to validate our nomograms. In the validation cohort, the area under the ROC curve reached 0.907 and 0.757 for PCR and GR, respectively, and the Brier scores for the calibration curves were 0.095 and 0.176, respectively (Fig. 5).

## Discussion

As far as we know, this study is the first to identify baseline PNI as an independent predictor of PCR, GR, and Response in ESCC patients receiving NCIT. Two new nomograms based on PNI predicted PCR and GR in ESCC patients well, but larger sample sizes are needed to validate our models in the future.

Previous studies have revealed that inflammation indexes were associated with the efficacy of neoadjuvant therapy in ESCC patients^24–26^. Zhang X et al.’s study showed that combining lymphocyte-to-monocyte ratio (LMR), SII with PLR could be considered a better predictor for discriminating responders and non-responders for ESCC patients receiving neoadjuvant anti-PD-1 plus chemotherapy^24^. Yang Y et al.’s study presented a novel predictor, the systemic inflammation-tumor markers index (SITI), for predicting pathologic complete response in resectable locally advanced ESCC patients receiving NICT^25^. Ma R et al.’s study evaluated factors affecting the objective response rate (ORR) and confirmed that aspartate aminotransferase (AST), D-dimer and carcinoembryonic antigen (CEA) were the independent predictors of ORR after neoadjuvant therapy with the TP regimen combined with PD-1 inhibitors for esophageal cancer^26^. While Zhang X et al. emphasized the combination of multiple markers, we found that PNI alone demonstrated significant predictive power, highlighting a simpler and potentially more accessible approach for clinical use. While Ma R et al. focused on the ORR, our study provides a comprehensive analysis of PCR, GR, and R, offering a more nuanced view of treatment response. We believe these differences provide further depth to offer practical clinical applications.

As one of the nutritional assessment indexes, PNI was first proposed by Buzby in the 1980s to predict the prognosis of patients following surgery for gastrointestinal malignancies^27^. Higher PNI predicted PCR in breast cancer patients receiving chemotherapy^28^longer PFS in head and neck squamous cell carcinoma patients receiving immunotherapy^29^and longer overall survival (OS) in patients with locally advanced nasopharyngeal carcinoma^30^. The body’s inflammatory state is strongly associated with cancer progression and immunotherapy efficacy^31^. Tumor-infiltrating lymphocytes (TILs) secrete various cytokines that shape the complex local immune microenvironment and exert both pro- and anti-tumor effects^32^. Kazuo Okadome reported that peripheral blood PNI in esophageal cancer patients was positively correlated with TILs in the microenvironment of cancerous tissues^33^. PNI and TILs are independent favorable prognostic markers for esophageal cancer patients, the latter associated with highly expressed PD1^34^, which explains the better pathological outcome of high PNI patients in our cohorts. Moreover, we investigated the correlation between PD-L1 expression and inflammatory Indexes. The significant negative correlations between PD-L1 and PIV/PLR/SII suggest that higher PD-L1 expression may reflect a less inflammatory peripheral immune profile. Conversely, PNI, which integrates albumin and lymphocytes, reflects nutritional and immune status rather than direct inflammation, explaining its lack of correlation with PD-L1.

In addition, we took the patient’s smoking history and neoadjuvant immunochemotherapy cycles into consideration. Smoking history remains a risk factor for the occurrence of esophageal cancer^35^. In our study, esophageal cancer patients who have never smoked were more likely to benefit from anti-PD1 immunochemotherapy. The PCR rate (6.8% vs. 23.2%), GR rate (30.1% vs. 41.9%), and response rate (54.8% vs. 58.1%) were lower in the smoking group than in the never-smoking group.

There were few studies on how cigarette smoke affected the microenvironment of esophageal cancer. Cigarette smoke enhances immunotherapy sensitivity by inducing PD-L1 expression in lung cancer^36^ but not in esophageal cancer^37^. In our study, smoking history was independently associated with pCR but not with GR or R. This discrepancy may reflect smoking’s complex immunomodulatory effects (e.g., suppressed cytotoxic lymphocytes and dendritic cell function)^37^which could selectively impair response mechanisms. “Cold” tumors are usually insensitive to anti-PD1 immunotherapy due to a lack of infiltrating CTLS with high PD1 expression^38,39^and the action of anti-PD-1 drugs requires the involvement of DCs to activate IL-12-driven antitumor response^40^. However, given the lack of association with broader response categories (GR/R), we emphasize that smoking should not be viewed as a definitive predictor but rather a factor warranting further investigation.

Surgery is usually scheduled after two to four cycles of NICT, with the clinician deciding whether to extend the number of rounds based on imaging changes and patient tolerance. A recent series of studies found that for patients with esophageal cancer, compared to two cycles of neoadjuvant therapy, three cycles of that significantly reduced T-stage^41^ and improved pathological response rate^42^ without increasing the risk of treatment-related adverse events (TRAEs) and death^43^. In addition, a recent prospective clinical study (NCT04459611) discovered that an additional course of sintilimab increased major pathological response (MPR) rate in NSCLC patients^44^. While cycles and deferred surgery (NICT-surgery interval) did not appear to affect immunotherapy response, this should still be a hot area for future investigation.

Our patient cohort received a variety of cytotoxic chemotherapeutic agents and different anti-PD-1 inhibitors, which could potentially confound the predictive value of the serum inflammation indexes we investigated. Firstly, the choice of chemotherapeutic agents could modulate the tumor microenvironment differently, affecting immune cell infiltration and cytokine release. Platinum-based regimens, for instance, are known to have immunomodulatory effects that may enhance the efficacy of immunotherapy. The inclusion of various chemotherapy drugs in our study might have introduced variability in the immunological response to anti-PD-1 therapy. Secondly, the use of different anti-PD-1 inhibitors which have distinct pharmacokinetic profiles and binding affinities could also impact the treatment outcome. Our study indicated a higher response rate with tirelizumab, although this observation requires validation in more homogenous settings. The heterogeneity in anti-PD-1 agents and chemotherapy regimens used could have introduced variability in the treatment response; however, the independence of PNI as a reliable predictor suggests its potential to stratify patients regardless of the treatment heterogeneity. It is important to note that while heterogeneity in treatment regimens could be a confounding factor, our study’s retrospective nature and the current standard of care, which often involves personalized treatment plans, make it challenging to control for this variability. Future studies with more stringent experimental designs, such as randomized clinical trials with limited chemotherapy regimens, are needed to further elucidate the impact of specific treatment regimens on the predictive value of serum inflammation indexes.

There are some limitations. Firstly, it was challenging to prevent measurement errors aroused by disparate equipment because our patients and their laboratory data were from two distinct hospitals. Secondly, limited by the sample size, our prediction model needs to be externally validated in a larger population. Moreover, since we excluded all patients receiving radiotherapy and other induction therapy, the predictive model’s utility in this patient population remained unknown. Finally, new experiments are optimized to balance confounding factors effect. Furthermore, single-cell sequencing could shed light on the genetic architecture and tumor immune environment in individuals with varying PNI levels. To confirm the prognostic importance of these factors, we will further improve the patient follow-up data in the future.

## Conclusions

In summary, we affirmed the vital role of baseline PNI in predicting the efficacy of neoadjuvant anti-PD-1 immunochemotherapy in ESCC patients. Simultaneously, based on our results and previous literature, smoking history and the number of NICT cycles deserve to be investigated. Given that this therapeutic paradigm is still in its preliminary stages, our findings will undoubtedly provide valuable guidance for clinical decision-making.

## Electronic supplementary material

Below is the link to the electronic supplementary material.

Supplementary Material 1

## Data Availability

The datasets used and/or analysed during the current study available from the corresponding author on reasonable request.
