# Supplementary material for: Predicting pathological response of resectable esophageal squamous cell carcinoma to neoadjuvant anti-PD-1 with chemotherapy using serum inflammation indexes
Source: Sci Rep. 2025 Jul 31;15:27914. doi: 10.1038/s41598-025-11590-x (PMC12313942; doi:10.1038/s41598-025-11590-x)
Supplement: Supplementary file 1 — Supplementary Material 1 [file 41598_2025_11590_MOESM1_ESM.docx]

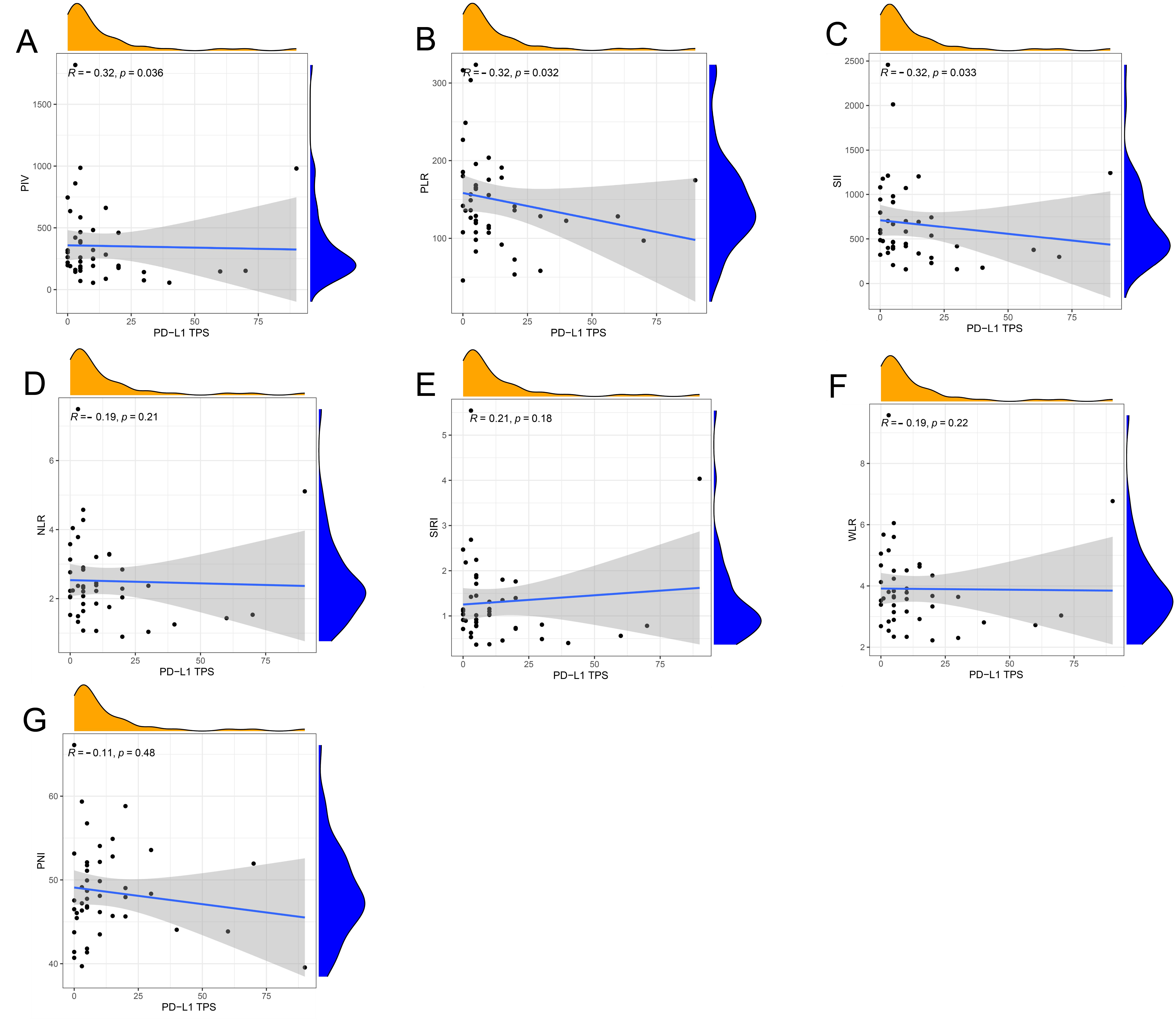


**Supplementary Figure 1.** The correlation between PD-L1 expression and inflammatory indexes.





**Supplementary Figure 2.** The prediction ability of statistically insignificant inflammatory indicators for PCR (A), GR (B), and R (C). PCR, pathologic complete response; GR, good response; R, response.
